# Mesenchymal Stromal Cell Secretome for Severe COVID-19 Infections: Premises for the Therapeutic Use

**DOI:** 10.3390/cells9040924

**Published:** 2020-04-09

**Authors:** Elia Bari, Ilaria Ferrarotti, Laura Saracino, Sara Perteghella, Maria Luisa Torre, Angelo Guido Corsico

**Affiliations:** 1 Department of Drug Sciences, University of Pavia, Viale Taramelli 12, 27100 Pavia, Italy; elia.bari@unipv.it (E.B.); sara.perteghella@unipv.it (S.P.); 2 Center for Diagnosis of Inherited Alpha1-antitrypsin Deficiency, Department of Internal Medicine and Therapeutics, Pneumology Unit IRCCS San Matteo Hospital Foundation, University of Pavia, 27100 Pavia, Italy; ilaria.ferrarotti@unipv.it (I.F.); corsico@unipv.it (A.G.C.); 3 Pneumology Unit IRCCS San Matteo Hospital Foundation, 27100 Pavia, Italy; l.saracino@smatteo.pv.it; 4 PharmaExceed S.r.l., 27100 Pavia, Italy

**Keywords:** SARS-CoV-2, COVID-19, acute respiratory distress syndrome, mesenchymal stem cells, extracellular vesicles, microvesicles, exosomes, secretome

## Abstract

From the end of 2019, the world population has been faced the spread of the novel coronavirus SARS-CoV-2 responsible for COVID-19 infection. In approximately 14% of the patients affected by the novel coronavirus, the infection progresses with the development of pneumonia that requires mechanical ventilation. At the moment, there is no specific antiviral treatment recommended for the COVID-19 pandemic and the therapeutic strategies to deal with the infection are only supportive. In our opinion, mesenchymal stem cell secretome could offer a new therapeutic approach in treating COVID-19 pneumonia, due to the broad pharmacological effects it shows, including anti-inflammatory, immunomodulatory, regenerative, pro-angiogenic and anti-fibrotic properties.

From the end of 2019, the World population has been faced with the spread of the novel coronavirus SARS-CoV-2 responsible for COVID-19 infection. SARS-CoV-2 is a positive single-stranded large RNA virus belonging to the B lineage of the *β*-coronaviruses. It is similar to the severe acute respiratory syndrome (SARS-1) and the Middle East respiratory syndrome (MERS) and it infects both humans and animals. The SARS-CoV-2 has a close similarity to bat coronaviruses [1] and its transition from animals to humans occurred in the Huanan seafood market in Wuhan, China, in December 2019 [2,3]. In just three months, the virus spread all over the World and on 11^th^ March 2020, the World Health Organization defined the COVID-19 as a pandemic. The clinical spectrum of the novel COVID-19 varies from asymptomatic or mild-disease (81% of all cases) to clinical conditions characterized by respiratory failure that requires mechanical ventilation (14% of all cases) and to systemic manifestations in terms of multiple organ dysfunction syndromes or failures (5% of all cases) [4] (Table 1).

The SARS-CoV-2 virus uses as a cellular entry the angiotensin-converting enzyme II (ACE2) receptor [1], which is widely distributed on the alveolar type II cells and capillary endothelium of the lungs, as well as in many other organs, including the cardiovascular, liver, kidney and gastrointestinal tract. It is probable that the lungs are particularly affected byCOVID-19 as they are the first organs to be infected and have a very slow turnover for regeneration. Huang and colleagues demonstrated that, after entering the cells, the virus could stimulate a terrible cytokine storm in the lung, increasing the levels of interleukin (IL)-2, IL-6, IL-7, granulocyte colony-stimulating factor (GSCF), interferon *γ*-induced protein 10 (IP10), monocyte chemoattractant protein-1 (MCP1), macrophage inflammatory protein (MIP1A) and tumor necrosis factor-alpha (TNF-α) [5]. The occurrence of the cytokine storm has been evidenced in many patients with COVID-19 pneumonia as well as by Wang and colleagues [6] and Leng and colleagues [7]. The pathologic data on the pneumonia caused by COVID-19 collected from autopsy or biopsy, despite being few at the moment, seem to confirm this hypothesis. Tian and colleagues first reported histopathological data obtained from the lungs of two patients who underwent lung lobectomies for adenocarcinoma [8]. Apart from the tumors, the pathologic findings in lung tissues were edema, prominent proteinaceous exudates (similar to those described in patients with the severe acute respiratory syndrome, SARS-1), hyperplasia of pneumocytes, vascular congestion and inflammatory clusters with fibrinoid material and multinucleated giant cells. The doctors retrospectively found that both patients were infected at the time of the surgery; therefore, these changes likely picture an early phase of the lung pathology of COVID-19 pneumonia.

At the moment, there is no specific antiviral treatment recommended for COVID-19. No vaccine is currently available. Antibacterial agents are ineffective. The therapeutic strategies are only supportive and oxygen therapy represents the primary treatment intervention for patients with severe pneumonia. Mechanical ventilation is necessary in cases of respiratory failure. The key to save the patients with severe COVID-19 pneumonia may be, in addition to inhibiting viral replication, preventing and reversing the cytokine storm. Systemic corticosteroids seem effective, but they also reduce the activity of the immune system [9] and thus its ability to fight against the infection. Moreover, the Italian Medicines Agency (AIFA, Agenzia Italiana del Farmaco) recently launched a clinical trial for tocilizumab, a monoclonal antibody against IL-6. However, the immunomodulatory capacity may be not strong enough, if only a few immune factors are used. It is our opinion that cellular therapies with mesenchymal stem cells (MSCs) could offer a new therapeutic approach. MSCs attract particular attention due to their broad pharmacological effects, including anti-inflammatory, immunomodulatory, regenerative, pro-angiogenic and anti-fibrotic properties [10]. 

Overall, plenty of preclinical studies have provided congruent and convincing evidence of MSC therapy effectiveness in treating many lung disorders. As recently reviewed, treatment with MSCs improved disease-associated parameters in acute respiratory distress syndrome (ARDS) [11] as well as bronchopulmonary dysplasia, chronic obstructive pulmonary disease, pulmonary hypertension and idiopathic pulmonary fibrosis [12,13,14,15]. To date, only two papers have considered MSCs for the treatment of COVID-19 pneumonia. According to Liang and colleagues, human MSCs from the umbilical cord effectively modulated the immune response and repaired the injured tissue of a 65-year-old female critically ill COVID-19 patient with excellent safety [16]. Clinicians administered the MSCs intravenously three times (5 × 10^7^ cells each time) every three days. After the second administration, the vital signs were improved, the trachea cannula was pulled off and the serum bilirubin, C-reactive protein (CRP) and alanine transaminase/aspartate transaminase (ALT/AST) were gradually reduced. Similarly, Leng and colleagues observed that MSC transplantation significantly improved the pulmonary function and symptoms of seven enrolled patients with COVID-19 pneumonia in two days [7]. In their study, 1 × 10^6^ cells per kilogram of weight were administered only one time. Authors demonstrated that the therapeutic effect was explicated mainly by the immunomodulating function of the MSCs. Notably, MSCs were ACE2 negative and thus, not infectable by the virus. Therefore, as suggested by Atluri and colleagues, MSCs could present a potential option for treating critically ill patients under compassionate use protocols [17].

Nevertheless, it is now evident from the literature that MSCs act by a paracrine mechanism. Indeed, these cells can be considered as potent drug stores releasing biologically active substances collectively known as secretome [18,19]. MSC-secretome is made of both soluble proteins, including a broad spectrum of cytokines, chemokines and growth factors and extracellular vesicles (EVs) of micro- and nano-size [20]. Once released, EVs and soluble proteins interact with the target cells (by ligand–receptor interaction or by internalization) and modulate cellular responses. In detail, secretome can activate endogenous stem cells and progenitor cells, suppress apoptosis, regulate the inflammatory response, stimulate the remodeling of the extracellular matrix and angiogenesis, reduce fibrosis and mediate the chemoattraction [21]. Therefore, as we reviewed recently [10], MSC-secretome emerges as a promising cell-free therapeutic tool for the treatment of acute and chronic lung diseases, as it displays the same anti-inflammatory, immunomodulatory, regenerative, pro-angiogenic and anti-protease properties of parental MSCs. Of note, unlike a monoclonal antibody, MSC-secretome acts on several cytokines simultaneously and potentially synergistically, as already reported in the literature on autoimmune diseases [22]. In the ARDS, the effectiveness of MSC-secretome in preclinical conditions is clear, both in vivo and ex vivo [23,24]. After intravenous injection, the secretome remained highly stable in the blood flow and distributed through to the lungs [25]. Here, secretome spread into the tissues and it provided immune modulation, resolution of inflammation, restoration of capillary barrier function and enhanced bacterial clearance [26].

Notably, the employment of secretome in therapy offers several advantages compared to MSCs. Secretome is generally considered safer than cells: it lacks the potential for endogenous tumor formation as it cannot self-replicate, it has low immunogenicity and when intravenously injected it leads to low emboli formation [27]. The use of MSC-secretome in therapy also entails technological advantages: it can be manipulated and stored more easily than cells, with fewer costs and it results in a ready to use product suitable for emergency interventions [10]. Finally, in comparison with monoclonal antibody therapy, the costs of MSC-secretome seem probably lower (tocilizumab costs $355.000 for a single dose [28]), which is important in treating a pandemic. These benefits become more evident for those countries, such as the majority of the African ones, where the handling of the COVID-19 outbreak is seriously challenged by the limited diagnostic and therapy capacities. For those counties, cell therapies cannot be implemented on-site due to the lack of adequate facilities and the delivery of cell preparations developed by industrialized countries, maintaining the cold chain, would be too expensive. In this regard, the possibility of pharmaceuticalizing MSC-secretome into freeze-dried and stable powder products would undoubtedly help in making this therapy more accessible. Recently, the literature reported evidence about the secretome “pharmaceuticalization” into a high quality, safe and effective medicinal product by exploiting large-scale and GMP preparation procedures [29,30,31]. This aspect is essential in providing physicians with sufficient amounts of standardized and ready-to-use products. Moreover, very recently, Bari and colleagues suggested, for the first time, that MSC-secretome can be formulated as inhalable dosage forms and as injectable dosage forms [10]. MSC-secretome administration by inhalation brings to profound clinical consequences. In essence, this route of administration provides a faster onset of action, allows lower doses to achieve the same effect as oral or injection therapy and being non-invasive, it avoids the side effects and pain typically associated with parenteral therapy. 

Based on these pieces of evidence, we hypothesize that the MSC-secretome, formulated as a freeze-dried powder and administered by intravenous injection (or inhalation), may represent a well suited approach for the treatment of patients with COVID-19 pneumonia, especially for the ones in critically severe condition. In this regard, two Chinese clinical trials recently appeared on http://www.clinicaltrials.gov (last access: 01/04/2020) investigating inhaled secretome for the treatment of COVID-19 pneumonia (NCT04276987) and its tolerance in healthy volunteers (NCT04313647). 

## Figures and Tables

**Table 1:** Clinical manifestations of the COVID-19 pandemic classified by the severity according to the Chinese National Health Commission.

Mild Disease	Severe Disease	Critical Disease
Fever; Respiratory symptoms; No pneumonia or mild pneumonia	Dyspnea; Respiratory frequency higher than 30/min; Blood oxygen saturation lower than 93% at rest state; PaO_2_/FiO_2_ ratio lower than 300 mmHg; Lung infiltrates higher than 50% within 24 to 48 h	Respiratory failure needs mechanical ventilation; Sepsis, septic shock; Multiple organ dysfunction or failure. Patients need Intensive Unit Care monitoring and treatment.
